# Relationship between vascular reactivity and expression of HMGB1 in a rat model of septic aorta

**DOI:** 10.1007/s00540-013-1584-x

**Published:** 2013-03-27

**Authors:** Satoshi Nishiike, Toshiaki Hiramatsu, Miharu Shiraishi, Yoshimichi Ueda, Hideaki Tsuchida

**Affiliations:** 1 Department of Anesthesiology and Perioperative Medicine, Kanazawa Medical University, Daigaku 1-1, Uchinada, Ishikawa, 920-0293 Japan; 2 Department of Pathology II, Kanazawa Medical University School of Medicine, Uchinada, Japan

**Keywords:** HMGB1, Vascular smooth muscle, Endothelium, Sepsis, Rat aorta

## Abstract

**Intruoduction:**

High mobility group box 1 (HMGB1), a ubiquitous nuclear protein, induces several inflammatory diseases and functions as a fatal factor when released extracellularly. The effect of HMGB1 on vascular reactivity during sepsis remains to be clarified.

**Methods:**

A rat model of abdominal sepsis was produced by cecal ligation and puncture (CLP) under sevoflurane anesthesia (*n* = 28). Anti-HMGB1 antibody at a dose of 4 or 0.4 mg/kg, or normal saline was injected twice intravenously, i.e., immediately after the CLP surgery and 4 h thereafter. Rats in the sham group underwent laparotomy, and the cecum was manipulated but not ligated or punctured. The descending thoracic aorta was excised 12 h after the CLP surgery and cut into rings of approximately 3 mm in length. Changes in the expression of HMGB1 and vascular reactivity were examined in the rings shortly after harvest and 4 h thereafter.

**Results:**

HMGB1 was identified immunohistochemically and by Western blotting in the nuclei of vascular endothelial and smooth muscle cells in all groups shortly after excision of the aorta, but its expression was augmented only in the CLP groups 4 h thereafter. Degenerated smooth muscle cells were also observed after CLP. Anti-HMGB1 antibody dose-dependently inhibited the augmentation of HMGB1 expression and the morphological changes induced by CLP. The expression of HMGB1 partly correlated with suppression of vascular reactivity.

**Conclusion:**

The present results strongly suggest that HMGB1 plays an important role in vascular malfunction from an early phase of sepsis.

## Introduction

High mobility group box 1 (HMGB1) is a ubiquitous nuclear protein present in many eukaryotic cells that stabilizes nucleosomes and enables gene transcription by binding to DNA [1]. When released to the extracellular space, however, HMGB1 induces several inflammatory diseases and functions as a late mediator, or as a fatal factor [2, 3]. During the early phase of sepsis, many proinflammatory cytokines, including tumor necrosis factor (TNF)-α and interleukin (IL)-1, are released by macrophages in response to endotoxin. HMGB1 is actively released by monocytes/macrophages more than 8 h after stimulation with endotoxin, TNF-α, or IL-1 [2, 4]. HMGB1 ligates three receptors expressed on the surface of endothelial and smooth muscle cells: the receptor for advanced glycation end products (RAGE), the toll-like receptor (TLR) 2, and TLR4 [5–7]. Endothelial cells stimulated with HMGB1 show increased expression of intercellular and vascular adhesion molecules and RAGE receptor, as well as increased secretion of TNF-α and chemokines, resulting in endothelial cell activation and injury [8]. HMGB1 and RAGE are known to regulate transendothelial migration of monocytes/macrophages [9], and cytoskeleton reorganization in smooth muscle cells [10].

It has been reported in the murine myocardium that cardiac dysfunction and HMGB1 expression are induced as early as 12 h after cecal ligation and puncture (CLP) surgery [11, 12]. Furthermore, glucan phosphate, a carbohydrate ligand that modulates innate immunity and proinflammatory signaling in sepsis, has been shown to improve cardiac function and HMGB1 translocation from the nucleus to the cytoplasm in the myocardium [11, 12]. These reports indicate that HMGB1 is not merely a late mediator but it is expressed in tissues from an earlier phase of sepsis and affects organ function. Cardiac dysfunction and vascular hyporeactivity are major consequences of severe sepsis and septic shock. It is well known clinically and experimentally that α-adrenergic vasoconstriction becomes attenuated during sepsis [13, 14]. Inducible nitric oxide synthase is expressed in septic vessels leading to an overproduction of nitric oxide [15]. Prostaglandins are also overproduced during sepsis [16], resulting in a negative inotropic effect on the heart [17] and hyporeactivity of blood vessels [18]. However, the role of HMGB1 regarding vascular reactivity has not been clarified, especially during the early phase of sepsis.

In this study, we used a rat model of CLP-induced sepsis to examine how HMGB1 influences vascular reactivity 12 and 16 h after peritonitis. We aimed at clarifying the relationship between vascular hyporeactivity and expression of HMGB1, both in the endothelium and vascular smooth muscle.

## Materials and methods

### Animals and protocol for induction of intraabdominal sepsis

This study was approved by the Animal Care and Use Committee of Kanazawa Medical University. All experimental procedures and handling of the animals were in accordance with the animal experiment guidelines of the National Institutes of Health Guide for the Care and Use of Laboratory Animals.

Male Sprague–Dawley rats weighing 200 and 250 g (*n* = 28) were used in the experiments. Intraabdominal sepsis was induced using the CLP technique (CLP group). Briefly, laparotomy through a midline abdominal incision was performed under sevoflurane anesthesia. An infrared heating lamp was used to prevent hypothermia throughout the surgery. The cecum was ligated with 3-0 ligature just below the ileocecal valve, so that intestinal continuity was maintained. The cecum was then punctured in two locations using an 18-gauge needle on the antimesenteric surface of the cecum, and was gently compressed until feces were slightly extruded. The bowel was returned to the abdomen and the incision was closed with a layer of proline sutures for the muscles and 3-0 silk for the skin. Rats in the sham operation group underwent laparotomy under sevoflurane anesthesia, and the cecum was manipulated but not ligated or punctured (sham group). All the animals were administered 60 ml/kg normal saline (NS) subcutaneously in their back after closure of the abdomen. Then, inhalation of sevoflurane was withheld, and the rats were observed in a recovery cage, deprived of food and water.

### Experimental protocol

In the CLP group rats, chicken anti-rabbit HMGB1 polyclonal antibody (Shino-test, Tokyo, Japan) at 4 mg/kg (CLP + 4 mgAb group; *n* = 7) or 0.4 mg/kg (CLP + 0.4 mgAb group; *n* = 7) in 1 ml NS or the same volume of NS (CLP + NS group; *n* = 7) was injected twice through the tail vein under light sevoflurane anesthesia immediately after the surgery and 4 h thereafter [19]. The sham group rats (*n* = 7) received only NS in a similar time course. The descending thoracic aorta was excised under deep sevoflurane anesthesia 12 h after the surgery, and placed in a cold, oxygenated (95 % O_2_:5 % CO_2_) physiological salt solution (PSS), with the following composition (in mM): NaCl, 118.3; KCl, 4.7; CaCl_2_, 2.5; MgSO_4_, 1.2; KH_2_PO_4_, 1.2; NaHCO_3_, 25.0; Ca-ethylenediaminetetraacetic acid (EDTA), 0.026; and glucose, 11.1. The aorta was gently cleaned of surrounding fat and connective tissue and cut into five rings approximately 3 mm in length. Then, two rings were incubated in an organ chamber filled with warm (37 °C, pH 7.4) oxygenated PSS for 4 h. The expression of HMGB1 in the aortic rings was analyzed by immunohistochemistry and Western blotting both shortly after preparing the aortic rings and after 4 h of incubation (two rings from each rat). For the immunohistochemical evaluation, the rings were fixed with 10 % buffered formaldehyde for 12 h at room temperature, dehydrated with a series of ethanol, and embedded in paraffin. To simultaneously localize the HMGB1 protein and detect macrophages in the rings, these were stained using anti-macrophage and anti-HMGB1 antibodies and examined under a fluorescence microscope. For the Western blotting analysis, two aortic rings were frozen in liquid nitrogen and stored at −80 °C until analysis.

Vascular reactivity was analyzed in the remaining ring. This aortic ring was suspended between two stainless steel wires in an organ chamber filled with 25 ml PSS (37 °C, pH 7.4) and gassed with a 95 % O_2_:5 % CO_2_ mixture. One of the wires was anchored in the organ chamber, and the other was connected to a strain gauge to measure isometric force. The aortic ring was progressively stretched and exposed to 40 mM KCl until the maximum contractile response was obtained. We used this maximum response to KCl as a reference tension. Then, phenylephrine (PE), an α_1_-adrenergic agonist, was administered cumulatively to the organ chamber to obtain final concentrations ranging from 10^–9^ M to 10^−5^ M. After washing the aortic ring with PSS several times, the ring was contracted with PE to cause approximately 60 % of the maximum PE-induced contraction (ED_60_). Then, acetylcholine (Ach), an endothelium-dependent vasodilator, was added cumulatively to the organ chamber to obtain final concentrations ranging from 10^−9^ M to 10^−5^ M. These procedures were repeated twice with an interval of approximately 4 h.

### Immunohistochemical procedures

The expression of HMGB1 was examined in aortic cross sections. Briefly, after 3-μm paraffin-embedded sections were deparaffinized in xylene, they were hydrated in a descending ethanol series and tap water. The sections were then incubated with 3 % H_2_O_2_ for 10 min to reduce endogenous peroxidase activity, and underwent antigen retrieval in 10 mM citrate buffer (pH 6.0) with 0.1 % Tween 20 for 15 min at 121 °C. Then, the sections were reacted with rabbit monoclonal anti-HMGB1 antibody (Epitomics, Burlingame, CA, USA) at a concentration of 0.75 μg/ml for 2 h at room temperature. The antibodies applied were visualized using the avidin–biotin–peroxidase technique (DAKO LSAB kit; DakoCytomation, Carpinteria, CA, USA) followed by chromogen detection with diaminobenzidine (DakoCytomation). The sections were counterstained with Mayer’s hematoxylin. The renal medulla was used as the positive control for HMGB1 because HMGB1 expression in renal tissue has been confirmed [20]. As the negative control, phosphate-buffered solution (PBS) was applied instead of the first antibody. Three sections were randomly selected from each animal to count the number of HMGB1-positive cells more than 250 μm in width.

### Double-labeled immunofluorescence staining

The sections were deparaffinized and underwent antigen retrieval the same as for immunohistochemistry. After blocking with 1 % bovine serum albumin (Wako, Osaka, Japan) for 30 min at room temperature, the sections were reacted with a mixture of rabbit monoclonal HMGB1 antibody (Epitomics) at a concentration of 10 μg/ml and mouse monoclonal anti-rat macrophage antibody (BMA-Biomedicals, Augst, Switzerland) at a concentration of 10 μg/ml for 2 h at room temperature. After washing with PBS, the sections were incubated for 1 h at room temperature with a mixture of Alexa Fluor 488-conjugated goat anti-rabbit IgG antibody (Invitrogen, Grand Island, NY, USA) diluted 1:200 and Alexa Fluor 594-conjugated goat anti-mouse IgG antibody (Invitrogen) diluted 1:200 in PBS. The sections were viewed with an LSM 710 Confocal Microscope (Carl Zeiss Japan, Tokyo) and were photographed digitally using LSM software ZEN 2010 (Carl Zeiss Japan).

### Western blotting analysis

After the rings were subsequently thawed in room air, homogenized in lysis buffer [50 mM Tris–HCl (pH 7.6), 10 % glycerol, 5 mM magnesium acetate, 0.2 mM EDTA, 1 mM phenylmethylsulfonyl fluoride, and 1 % sodium dodecylsulfate], and centrifuged for 10 min at 15,000 *g*, at 4 °C. The supernatant was collected, and the protein (10 μg) was applied to and electrophoresed on a 10 % polyacrylamide gel and transferred to a nitrocellulose membrane (Atoh, Tokyo, Japan). The membrane was reacted overnight at 4 °C with rabbit monoclonal anti-HMGB1 antibody (Epitomics) at a concentration of 1 μg/ml. After incubation with peroxidase-labeled goat anti-rabbit IgG antibody (DakoCytomation) for 1 h at room temperature and vigorous washing, the nitrocellulose membrane was incubated with Immuno Star Kit (Wako) and photographed digitally using Image Quant LAS 4000 (GE Healthcare Japan, Tokyo). All samples were standardized by immunoblot using anti-β-actin mouse monoclonal antibody (Sigma, St. Louis, MO, USA).

### Measurement of vasoreactivity

To observe vascular response, we used the PowerLab organ bath system (AD Instruments Japan, Nagoya), and vascular tension was continuously monitored with the Chart software v3.6.1 (AD Instruments Japan).

### Statistical analysis

Results are presented as the mean ± standard deviation. For continuous variables, the Student *t* test was used for comparison between two groups and one-way analysis of variance (ANOVA) for multiple comparisons with the post hoc Fisher’s LSD test. For categorical variables, Mann–Whitney *U* test was used for comparison between two groups and Kruskal–Wallis test for multiple comparisons. If a significant difference was obtained using the Kruskal–Wallis test, Mann–Whitney *U* test with Bonferroni correction was used for post hoc analysis. A *P* value less than 0.05 was considered statistically significant.

## Results

**Fig. 1:**
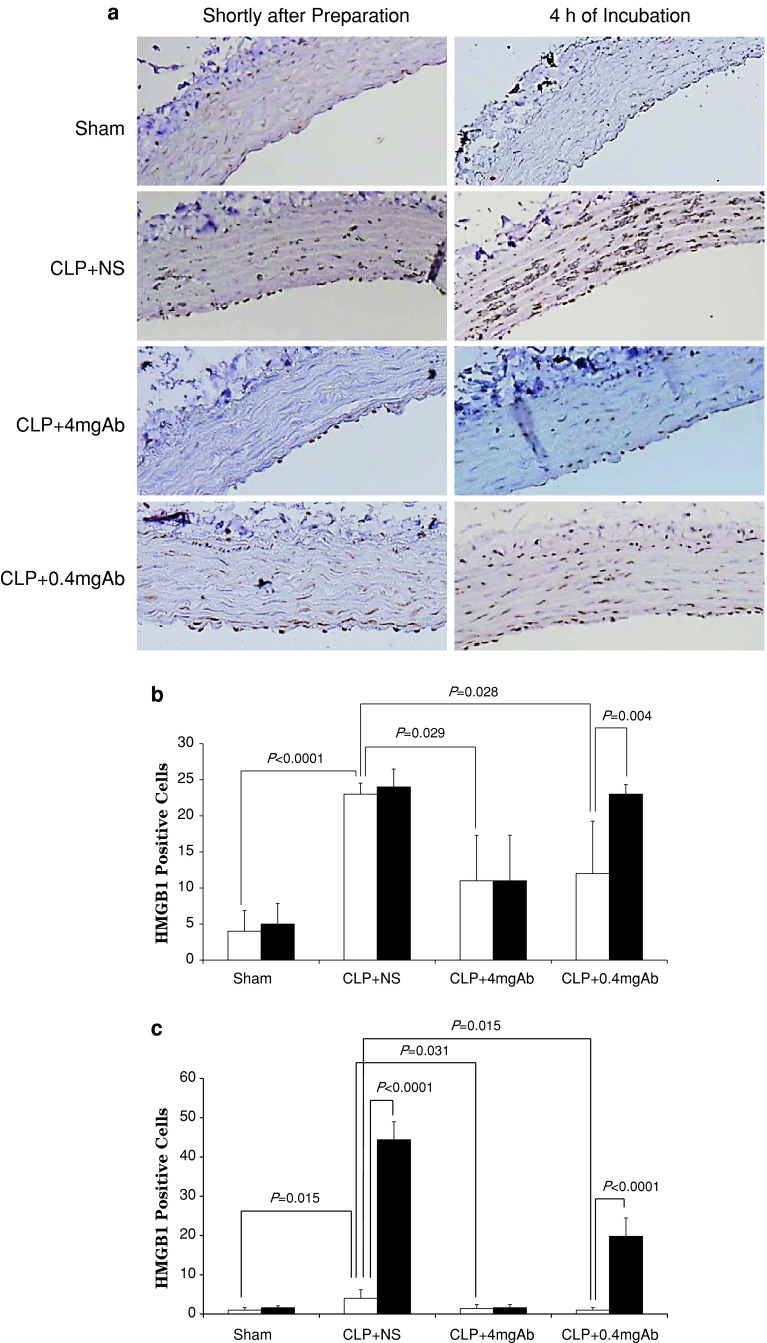
**a** Immunohistochemical imaging of a rat aortic section. ×100. *Dark brown* indicates high mobility group box 1 (HMGB1) protein. **b** Number of immunohistochemically defined HMGB1-positive endothelial cells. **c** Number of immunohistochemically defined HMGB1-positive smooth muscle cells in the four groups. *White columns* indicate the strip shortly after preparation; *black columns* indicate the strip after 4 h incubation. Each, *n* = 7

Figure 1a shows the immunohistochemical findings in the aortic rings in the four groups. Although HMGB1 was expressed in the nuclei of the endothelium in all groups even shortly after the preparation, the number of HMGB1-positive endothelial cells in the CLP groups (23 ± 2 in the CLP + NS group, 11 ± 6 in the CLP + 4 mgAb group, and 12 ± 7 in the CLP + 0.4 mgAb group) was significantly greater than that in the sham group (4 ± 3; Fig. 1b). The number of HMGB1-positive endothelial cells did not change significantly in aortic rings examined 4 h after the preparation in the sham (6 ± 3), CLP + NS (24 ± 1), and CLP + 4 mgAb (12 ± 6) groups. In contrast, the number of HMGB1-positive endothelial cells was significantly increased at this time point in the CLP + 0.4 mgAb group (23 ± 1). As shown in Fig. 1c, HMGB1 was also expressed in the nuclei of smooth muscle cells; 1 ± 1 in the sham group, 4 ± 2 in the CLP + NS group, 1 ± 1 in the CLP + 4 mgAb group, and 1 ± 1 in the CLP + 0.4 mgAb group. The number of HMGB1-positive smooth muscle cells was significantly greater in the CLP + NS group than that in the sham group shortly after the preparation. The number of HMGB1-positive smooth muscle cells was increased at the 4-h time point and reached a statistically significant level as compared to that shortly after the preparation in the CLP + NS (44 ± 5) and CLP + 0.4 mgAb (20 ± 5) groups, whereas it did not change significantly in the sham (2 ± 0) and CLP + 4 mgAb (2 ± 1) groups.

In addition to HMGB1 expression, degenerated smooth muscle cells with dark-stained cytoplasm could be observed only in the CLP + NS group. The number of degenerated smooth muscle cells was increased after 4 h incubation compared with that shortly after the preparation (31 ± 21 and 113 ± 39 at shortly after preparation and 4 h thereafter, respectively; *P* = 0.016). Furthermore, in the sham group smooth muscle cells were all lined in a wavy fashion shortly after the preparation and 4 h thereafter. In contrast, in the CLP + NS group smooth muscle cells were all lined straight both shortly after the preparation and 4 h thereafter (*P* = 0.0025 vs. the sham group). In the CLP + 4 mgAb group, smooth muscle cells were lined wavily in all rings shortly after the preparation (*P* = 0.0025 vs. the CLP + NS group), whereas they were lined straight in 2 of 7 rings 4 h thereafter. In the CLP + 0.4 mgAb group, although smooth muscle cells were lined wavily in 6 of 7 rings shortly after the preparation (*P* = 0.014 vs. the CLP + NS group), they were lined straight 6 of 7 rings 4 h thereafter (*P* = 0.037 vs. shortly after the preparation). These results indicate that morphological changes develop as HMGB1 expression increases in smooth muscle cells after CLP.

**Fig. 2:**
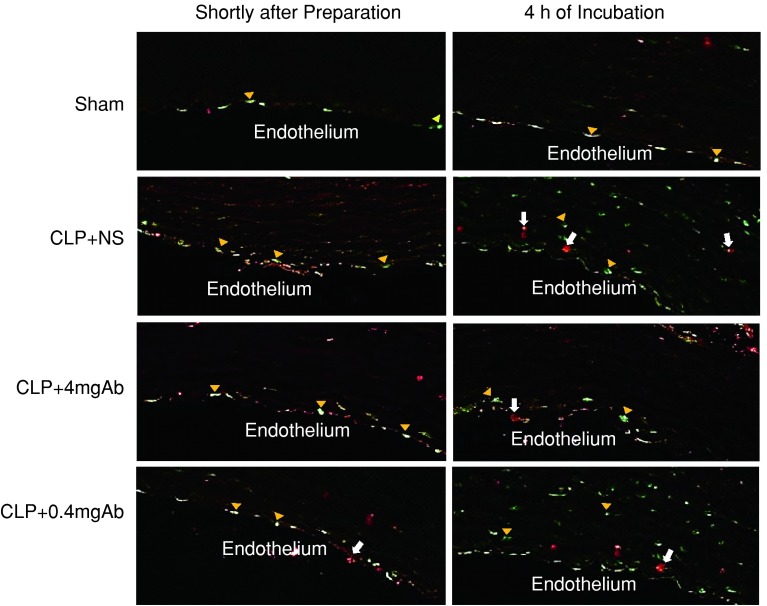
Double-labeled immunofluorescence imaging of an aortic section. ×400. *Green* indicates HMGB1 protein; *red* indicates macrophages. In the sham group, HMGB1 protein expression (*yellow arrowheads*) was restricted to the endothelium shortly after the preparation and 4 h thereafter. No macrophage (*white arrows*) was observed in the vascular slices. In contrast, HMGB1 protein was expressed in the endothelium and macrophages were attached to it in the NS group shortly after the preparation. The expression of HMGB1 protein extended to the nuclei of smooth muscle cells and macrophages were identified in the smooth muscle layer 4 h thereafter. The changes in expression of HMGB1 protein and macrophage migration observed in the NS group were almost completely inhibited in the 4 mgAb group, whereas they were partly suppressed in the 0.4 mgAb group. *CLP* cecal ligation and puncture; *NS* normal saline

Figure 2 shows double-labeled immunofluorescence staining using anti-HMGB1 and anti-macrophage antibodies. In the sham group, HMGB1 protein expression (green) was restricted to the endothelium shortly after the preparation and 4 h thereafter. No macrophages were observed in the vascular slices in the sham group. To the contrary, HMGB1 protein was expressed in the endothelium and macrophages were attached to it in the CLP + NS group shortly after the preparation. The expression of HMGB1 protein was spread into the nuclei of smooth muscle cells 4 h thereafter. In addition, macrophages were identified in the smooth muscle layer in the CLP + NS group 4 h after the preparation. The changes in the expression of HMGB1 protein and macrophage migration observed in the CLP + NS group were almost completely inhibited in the CLP + 4 mgAb group, whereas they were partly suppressed in the CLP + 0.4 mgAb group.

**Fig. 3:**
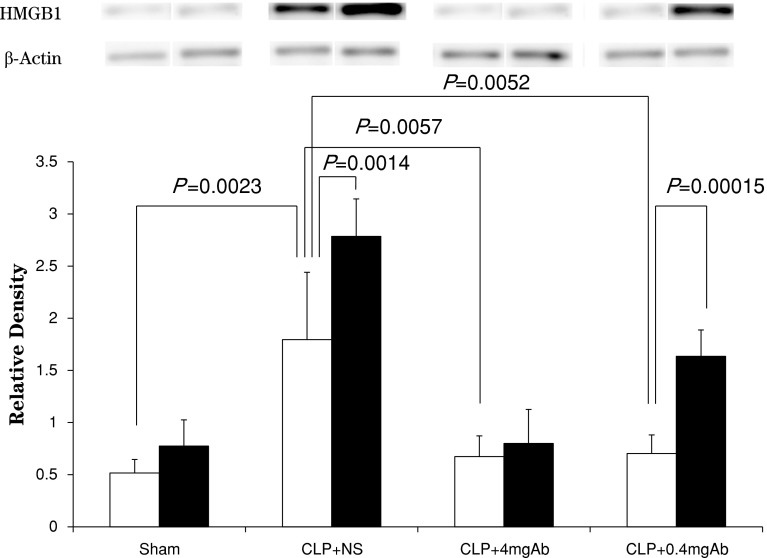
Expression of HMGB1 analyzed by Western blotting in the four groups. The expression is presented as density relative to that of β-actin. *White columns* indicate the strip shortly after preparation; *black columns* indicate the strip after 4 h incubation. Each, *n* = 7, each. See text for details

The results of Western blotting analysis of HMGB1 in the aortic rings are shown in Fig. 3 as density relative to that of β-actin. Expression of HMGB1 shortly after preparation of the rings was significantly greater in the CLP + NS group than in the other three groups. Although the expression of HMGB1 increased during incubation for 4 h as compared to that shortly after the preparation, only in the CLP + NS and CLP + 0.4 mgAb groups it reached levels of statistical significance.

**Fig. 4:**
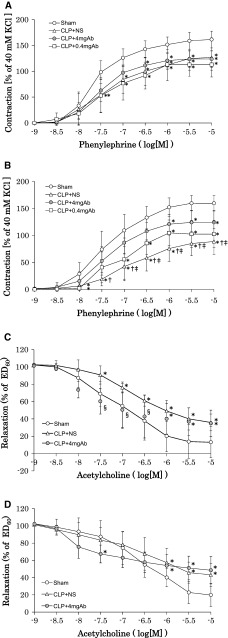
*Left* Phenylephrine-induced vascular contraction in the four groups (**a**, **b**). Reference tension (100 %) was obtained with 40 mM KCl before phenylephrine challenge. **a** Aortic ring shortly after preparation. **b** Aortic ring 4 h thereafter. *Right* Acetylcholine-induced vasodilation in the sham, CLP + NS, and CLP + 4 mgAb groups (**c**, **d**). The CLP group administered CLP + 0.4 mgAb was omitted from the calculation because acetylcholine-induced vasodilation was inconsistent from ring to ring. The ring was preconstricted with phenylephrine to obtain approximately 60 % of the maximum contraction (ED_60_). **c** Aortic ring shortly after preparation. **d** Aortic ring 4 h thereafter. Data are expressed as the mean ± standard deviation. Each, *n* = 7. *P < 0.05 versus the sham group at the respective time point, ^†^ *P* < 0.05 versus the ring shortly after the preparation in the same group, ^‡^ *P* < 0.05 versus the 4 mgAb group at the same time point, ^§^ *P* < 0.05 versus the NS group at the respective time point

PE-induced vascular contractions are depicted in Fig. 4a, b. As compared to the sham group, PE-induced contraction was significantly attenuated in the CLP groups irrespective of the administration of anti-HMGB1 antibodies in the first series (Fig. 4a). There were no significant differences in contractile response among the CLP groups. In the second series performed 4 h after the first one (Fig. 4b), PE-induced contraction was comparable to the first one in the sham group, as well as in the CLP groups administered anti-HMGB1 antibodies (*P* = 0.81, 0.90, and 0.42 for the sham, CLP + 4 mgAb, and CLP + 0.4 mgAb groups, respectively). In contrast, PE-induced contraction was further attenuated in the second series compared to the first one in the CLP + NS group (*P* = 0.043). As a result, a significant difference in the PE-induced contraction was observed between the CLP + 4 mgAb group and the CLP + NS group (*P* = 0.017).

Ach-induced vasodilation is shown in Fig. 4c, d. Ach-induced vasodilation was inconsistent and showed remarkable variation among the aortic rings in the CLP + 0.4 mgAb group. We excluded this group from data analysis, and Ach-induced vasodilation was examined only in the sham, CLP + 4 mgAb, and CLP + NS groups. Ach dose-dependently relaxed the rings preconstricted with PE in the three groups in both the first and second series. Ach at the dose of 10^−5^ M caused maximum endothelium-induced vasodilation of approximately 80–90 % of preconstriction in the sham group. In the CLP groups, however, maximum vasodilation was attenuated as compared to the sham group (*P* < 0.05). These results indicate that abdominal sepsis inhibits not only PE-induced vasoconstriction but also endothelium-induced vasodilation, both of which were partly restored by anti-HMGB1 antibody.

## Discussion

In the present study, we demonstrated that HMGB1 was expressed in the endothelium of the descending thoracic aorta 12 h after CLP surgery and that 4 h later it was also expressed in smooth muscle cells. Moreover, we showed that morphological changes became apparent when HMGB1 expression was detected in smooth muscle cells, and these changes were partly reversed by anti-HMGB1 antibody. Besides, both PE-induced vasoconstriction and Ach-induced endothelium-dependent vasodilation were attenuated in the thoracic aorta 12 h after CLP surgery, and this hyporeactivity was even more marked at 4 h thereafter (second series). In addition, anti-HMGB1 antibody administered after CLP surgery partially but dose-dependently reversed the progression of HMGB1 expression and the attenuation of vascular responses. These results strongly indicate that HMGB1 is a mediator responsible for vascular inflammation and hyporeactivity in sepsis.

HMGB1 is induced in patients with inflammatory diseases, and the serum concentration of HMGB1 is increased during peripheral tissue insult [21–24]. Therefore, it is not surprising that HMGB1 was expressed in the endothelium even after the sham surgery. The most striking findings of the present study were that the expression of HMGB1 increased and macrophages migrated to the smooth muscle layer only in the CLP group of the ex vivo experiment, when neither HMGB1 nor proinflammatory cytokines were present in the medium around the rings. In addition, anti-HMGB1 antibody administered in vivo partly but dose-dependently neutralized the morphological and functional changes observed in aortic rings from rats challenged by CLP. These results indicate that the endothelium was activated as early as 12 h after CLP surgery, resulting in leukocyte adherence and loss of the barrier function. This observation is consistent with the findings reported by Susa et al. [19], who showed that serum HMGB1 levels peaked at 12 h after CLP surgery, and those reported by Ha et al. [11, 12], who found that cardiac dysfunction and HMGB1 expression were induced 12 h after CLP surgery.

To our best knowledge, this is the first report showing the expression and significance of HMGB1 in the rat aorta during sepsis. Furthermore, HMGB1 expression seemed to be limited to the nuclei of endothelial and smooth muscle cells in this study. It has been shown that HMGB1 is expressed in smooth muscle cells underlying atherosclerotic lesions [25, 26]. However, in those studies, most HMGB1 immunoreactivity was observed in the cytoplasm as compared to that in the nucleus. Thus, our findings together with those of Kalinina et al. [25] and Inoue et al. [26] suggest that HMGB1 expression is augmented but restricted to the nucleus of smooth muscle cells during the early phase of inflammation, whereas in the chronic inflammatory status, HMGB1 is also expressed in the cytoplasm.

Several mechanisms have been advocated to induce vascular hyporeactivity during sepsis [13–16, 18]. In the present study, HMGB1 expression in aortic rings showed a fair and negative correlation with both PE-induced contraction and Ach-induced endothelium-mediated vasodilation. The present study did not clarify the exact mechanisms by which expression of HMGB1 in the nucleus produced vascular hyporeactivity during sepsis. Although in previous studies the exact mechanisms were not delineated either, the relationship between HMGB1 expression and organ injury was demonstrated in the lungs [27, 28], heart [11, 12], and diaphragm [19]. Administration of anti-HMGB1 antibody was effective to alleviate both sepsis-induced and non-sepsis-induced organ injury [21, 24, 29]. Degryse et al. [10] have reported that HMGB1 not only stimulates migration of rat smooth muscle cells by chemotaxis, but also induces rapid and transient changes of the cell shape and actin cytoskeleton reorganization, leading thereby to an elongated polarized morphology. In this study, morphological changes in smooth muscle cells coincided with HMGB1 expression and vascular malfunction, indicating that HMGB1 expression has a key role in morphological changes and vascular hyporeactivity.

There were significant differences between the sham and CLP groups in terms of smooth muscle contraction, but HMGB1 expression in smooth muscle cells was comparable among the sham, CLP + 4 mgAb, and CLP + 0.4 mgAb groups shortly after the preparation. Therefore, HMGB1 expression did not exactly correlate with vascular contraction. This result indicates that HMGB1 expression is not the sole factor inducing hyporeactivity of the aorta during sepsis. Other factors including activated macrophages [6] attached to the endothelium and overproduction of nitric oxide [13–15] and prostaglandins [16, 18] may account for this hyporeactivity.

There are several drawbacks in our experiment. We did not measure the serum levels of HMGB1 or other cytokines. We do not know how anti-HMGB1 antibody influenced those cytokine levels. However, our results agreed very well with those of previous studies showing that CLP surgery induced HMGB1 expression at tissue level and that anti-HMGB1 antibody effectively reversed this alteration [19, 30]. Rat aortae were harvested 12 h after the surgery and were immersed in PSS without HMGB1 or cytokines, which should have influenced the results. We suppose, however, that the alteration would have been more accelerated and intense than the one we observed when the rings were in situ. We postulated 40 mM KCl-induced tension as a reference contraction shortly after excision of the aortae and did not measure the absolute force of the aortic rings. Thus, KCl-induced contraction could have been attenuated even at 12 h after surgery in the CLP groups. Yet, if this were true, the differences in muscle tension between the sham group and the CLP groups and among the CLP groups should have been more obvious.

In conclusion, CLP surgery induced HMGB1 expression in the rat aorta, which was accompanied by morphological and functional derangement. Anti-HMGB1 antibody could partly reverse these alterations after CLP. Expression of HMGB1 in the endothelium and smooth muscle cells could be another cause of hemodynamic perturbation even from an early phase of severe sepsis.
